# Detection of protein symmetry and structural rearrangements using secondary structure elements

**DOI:** 10.1002/pro.70576

**Published:** 2026-04-30

**Authors:** Runfeng Lin, Sebastian E. Ahnert

**Affiliations:** ^1^ Theory of Condensed Matter Group, Cavendish Laboratory University of Cambridge Cambridge UK; ^2^ Department of Chemical Engineering and Biotechnology University of Cambridge Cambridge UK; ^3^ The Alan Turing Institute London UK

**Keywords:** computational biology, protein symmetry, protein secondary structure, protein structure

## Abstract

Many proteins exhibit a degree of internal symmetry in their tertiary structure, including circular permutations. These characteristics play an important role in terms of the functional robustness of proteins against mutations, and are pivotal for the study of protein function and evolution. Proteins exhibiting internal symmetry often demonstrate enhanced functional benefits, such as increased binding affinity due to repeated structural motifs, and evolutionary advantages that may result from gene duplication or fusion events, leading to more robust and adaptable molecular architectures. Similarly, circular permutations have been linked to improved catalytic activity and enhanced thermostability, opening new avenues in the design of enzymes and other functional proteins. In this study, we introduce a novel computational pipeline that leverages secondary structure elements (SSEs) as a compressed yet informative representation of protein structure to detect both symmetry and structural rearrangements effectively. Our method outperforms existing methods, such as Combinatorial Extension with Circular Permutations (CECP) by several orders of magnitude in terms of computational cost and identifies 17,130 circularly related proteins. Additionally, it detects 26,739 proteins associated with indel mutations. Notably, 8855 of these exhibit both circular permutation and indel mutations—highlighting a significant structural co‐occurrence between these two types of variation. We also recovered most symmetric proteins identified by the CE‐Symm tool and revealed more than 2.5 times as many symmetric proteins in the same dataset. By integrating precise boundary detection, clustering analysis, and rigorous cross‐validation against established tools, our framework robustly maps internal structural features. These insights deepen our understanding of protein architecture and provide a structural basis for further investigation into protein evolution, while also informing de novo protein design and engineering.

## 1 INTRODUCTION

Proteins fulfill a myriad of essential functions in every cell, and their three‐dimensional (3D) structures underpin these functional roles. The tertiary structure of a protein is determined by the intricate interplay between amino acid interactions and environmental influences. While significant progress has been made in predicting protein structures (Jumper et al., 2021) and simulating protein dynamics (Wang et al., 2024), many structural studies have primarily focused on global folding patterns, often overlooking finer internal characteristics such as symmetry, indel mutations, and circular permutations (Bliven et al., 2014; Lo et al., 2008).

Research has demonstrated that symmetric and simplified structural motifs are intrinsically more likely to emerge in evolutionary processes (Johnston et al., 2022). Internal symmetry in proteins is more than a mere structural curiosity; it has profound functional implications. Proteins with symmetrical architectures can exhibit increased binding affinities due to the presence of multiple identical binding sites. Furthermore, they may benefit from enhanced stability and robustness, traits historically linked to evolutionary events like gene duplication or fusion (Tom & Srinivasan, 1996).

The vast majority of proteins do not function as isolated monomers; instead, they operate as complexes. Most of these are homomeric, forming symmetric quaternary structures (Therese Bergendahl & Marsh, 2017; Villar et al., 2009). The specific symmetry groups formed by these homomers significantly dictate their function, providing evolutionary advantages that include enhanced thermodynamic stability, optimized surface‐to‐volume ratios, and the efficient formation of allosteric networks (Therese Bergendahl & Marsh, 2017). Furthermore, many internally symmetric proteins are the evolutionary descendants of these ancient homomers. Following events of gene duplication and fusion, a symmetric multimeric complex can consolidate into a single continuous polypeptide chain (Levy & Teichmann, 2013; Tom & Srinivasan, 1996). Internally symmetric proteins formed via this evolutionary pathway preserve the structural and functional benefits of their symmetric ancestral origins while operating at a lower entropic cost (Blaber et al., 2012).

Circular permutations, wherein the protein chain is rearranged so that the original termini are reconnected at new positions, have been associated with increased catalytic efficiency and improved thermostability (Cheltsov et al., 2001; Topell et al., 1999). Insertions and deletions (indels) are a crucial force in protein evolution. By subtly altering the length and flexibility of loop regions, indels can modify protein function—affecting binding interactions, enzyme specificity, and overall stability. Although typically small, these changes offer a mechanism for evolutionary innovation, allowing proteins to adapt new functions while preserving their essential structural framework (Pascarella & Argos, 1992). Such rearrangements suggest that nature exploits these features to fine‐tune protein function and adaptability.

**FIGURE 1:**
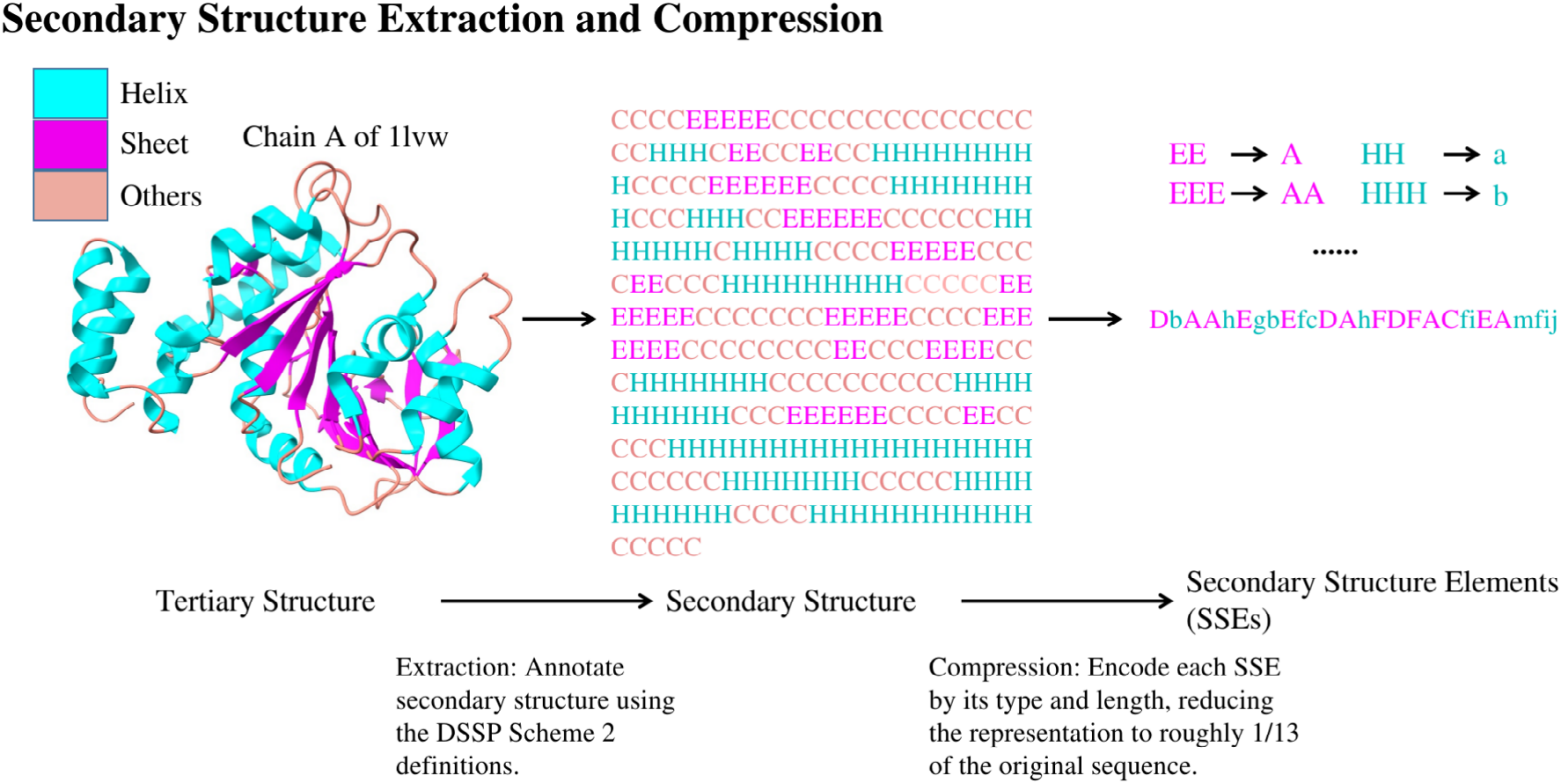
Extraction of secondary structure elements (SSEs). Secondary structures are initially assigned from the tertiary structure using DSSP (Joosten et al., 2011) (following Scheme 2). These assignments are subsequently compressed into SSEs by aggregating contiguous segments of helices and sheets. In the final encoding, sheets are represented by uppercase letters and helices by lowercase letters (Lin & Ahnert, 2025).

Seminal work by the Rose group has established that secondary structure (SS) organization alone contains sufficient information to distinguish protein folds, often outperforming primary structure comparisons in classification tasks. (Przytycka et al., 1999) Building on this, Gong and Rose developed a finer‐grained representation of local structure—termed “mesostates”—to achieve high‐fidelity protein fold recognition. (Gong & Rose, 2005) Crucially, the determinative power of secondary structure extends beyond classification to physical folding. Implementation of these coarse‐ and fine‐grained constraints within Monte Carlo simulations, augmented only by generic hydrogen bonding and steric potentials, proved that defined secondary structure patterns effectively restrict the conformational search space, guiding the polypeptide backbone to its unique native topology. (Fleming et al., 2006; Gong et al., 2005) Building on these biophysical principles, our recent work demonstrates that the rigorous constraint between secondary and tertiary structure is preserved even when the representation is further compressed. We introduced Secondary Structure Elements (SSEs)—sequences composed solely of consecutive helices and sheets—as an ultra‐compact encoding of protein architecture (Figure 1; Section 4.1). By focusing exclusively on SSEs, we reduce the complexity inherent in residue‐ or atomic‐level representations while retaining the critical topological information defining the global fold (Lin & Ahnert, 2025). Our encoding is a more compact representation than the 3Di alphabet employed by Foldseek (van Kempen et al., 2023), which encodes tertiary interactions into residue‐level sequences in order to accelerate structure search. This highly informative representation has since been widely adopted in downstream tasks, ranging from the development of structure‐based protein language models (Heinzinger et al., 2024) to the detection of circular permutations (Ding et al., 2025).

In this work, we used SSEs to develop a pipeline that detects protein symmetry, circular permutations, and indel mutations. Our approach begins with the extraction of SSEs, followed by precise boundary detection and a clustering algorithm designed to capture subtle rearrangements. The pipeline is validated against established databases and benchmarked against state‐of‐the‐art tools, ensuring that our method is both reliable and sensitive to variations in protein structure. In terms of speed, our SSE‐based method significantly outperforms the Combinatorial Extension with Circular Permutations (CECP) (Bliven et al., 2014), which is more than 16,000 times slower. Our approach uncovers 17,130 circularly related proteins. Notably, over 8855 of these are both circularly permuted and exhibit evidence of indel mutations. This suggests that circular permutations may often occur in tandem with indel mutations. We also recover most of the symmetric proteins that can be identified using the CE‐Symm tool (Bliven et al., 2019) and reveal in total more than 2.5 times as many symmetric proteins in the same dataset, underscoring the sensitivity and breadth of the pipeline.

## 2 RESULTS

### 2.1 SSE duplication pipeline

In order to efficiently detect internal symmetries and structural rearrangements, we developed a computational pipeline based on Secondary Structure Elements (SSEs). Unlike residue‐level representations (such as amino acid sequences or 3Di) that maintain a one‐to‐one mapping with the backbone, our approach compresses the protein topology by aggregating contiguous residues into single functional units.

Specifically, secondary structures are assigned using DSSP (Scheme 2) (Joosten et al., 2011), where consecutive residues of the same type are grouped into a single element. In this encoding, β‐strands are represented by uppercase letters (e.g., “A,” “B”) and α‐helices by lowercase letters (e.g., “a,” “b”), while coil regions are excluded to focus on the rigid topological framework. More details are in Section 4.1.

This abstraction yields a representation approximately 9.85‐fold more compact than the original amino acid sequence in terms of Shannon entropy. This significant reduction in sequence length converts the computationally expensive $O\left(L^{2}\right)$ residue‐alignment problem into a rapid element‐alignment task.

**FIGURE 2:**
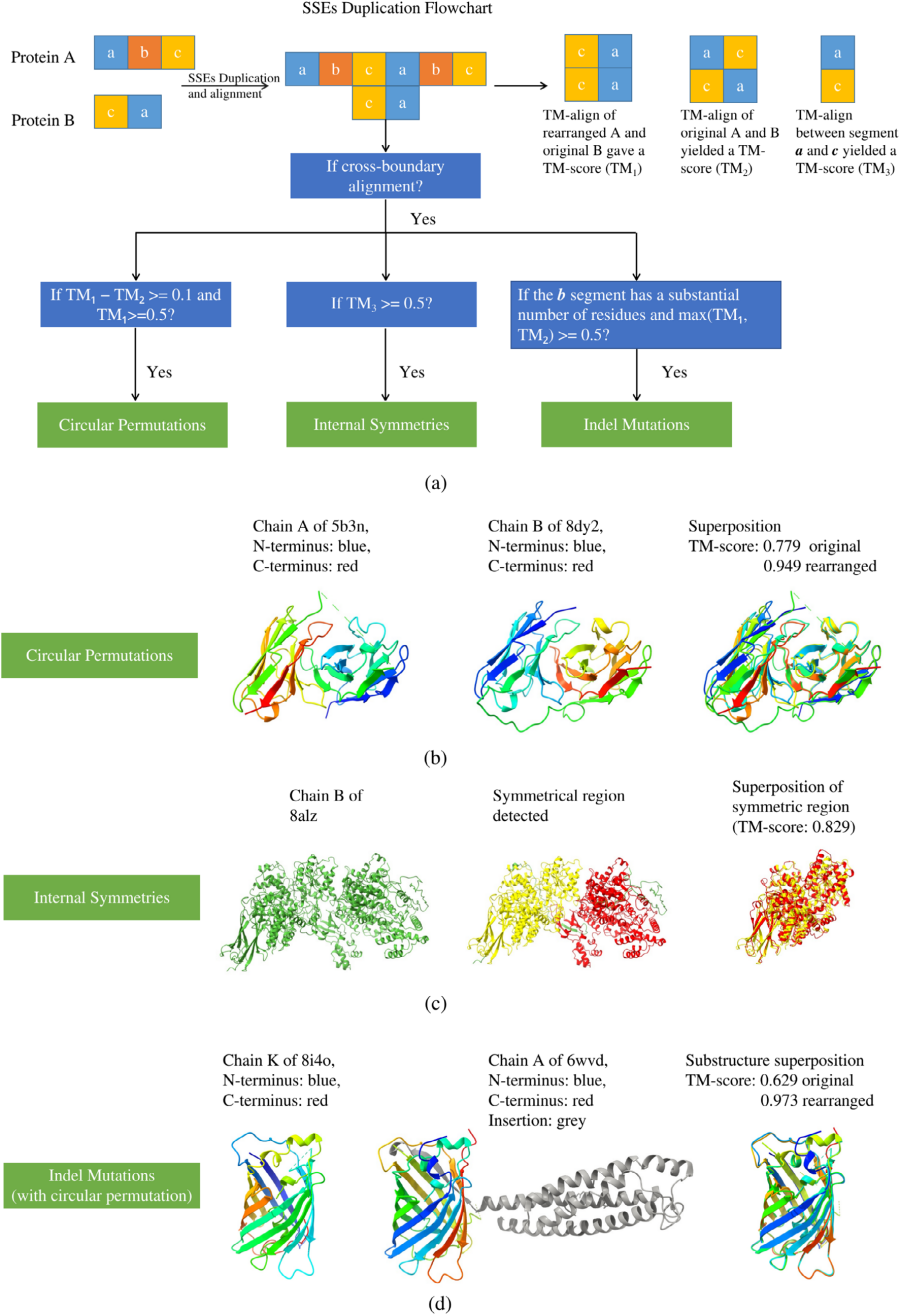
SSE duplication algorithm and performance on challenging cases. (a) Workflow of the SSE duplication pipeline. (b) Circular Permutation: Application to a CP instance (5b3n_A‐8dy2_B) that was classified as a non‐match by CECP. (c) Internal Symmetry: Identification of symmetry in (8alz_B) which was not found by CE‐Symm. (d) CP with Indels: Robust detection of circular permutation accompanied by significant indel mutations (8i4o_K‐6wvd_A), a scenario where standard CECP alignment failed. These examples highlight the pipeline's capability to uncover structural similarities in outliers missed by conventional methods.

Using this compact encoding, we search for structural duplications via adjusted Smith‐Waterman alignment. We evaluated this SSE‐duplication pipeline on a non‐redundant subset of the RCSB PDB, comprising 71,781 protein chains (sequence identity ≤90%, length ≥5 SSEs). This filtering minimizes bias and ensures broad structural coverage. Figure 2a summarizes the three main steps: SSE duplication, precise boundary detection, and validation (Figures S6 and S8).

#### 2.1.1 Detection of circular permutations

Applying the pipeline to this dataset, we identified 67,984 circularly permuted pairs across 17,130 unique proteins (Figure 2b). Leiden clustering (Traag et al., 2019) grouped these proteins into 453 communities, with 70% of members concentrated in the 10 largest clusters—indicating a highly skewed distribution.

For external validation, we assessed the performance of SSE duplication against the Combinatorial Extension with Circular Permutations (CECP) (Bliven et al., 2014) method using the same dataset. As CECP is computationally intensive—requiring an estimated 2.2 million CPU hours for full‐scale analysis—we restricted the comparison to the 67,984 pairs detected by SSE duplication. In addition, we used the Circular Permutation DataBase (CPDB) as a representative benchmark set and compared how well CECP and SSEs duplication can reproduce the results.

*Comparison with CECP* – Among the 67,984 pairs analyzed using CECP, only 40,058 had a TM‐score above 0.5, and not all were recognized as circular permutations. In contrast, SSE duplication requires both a post‐rearrangement TM‐score above 0.5 and an improvement of at least 0.1 over the original to qualify as a circular permutation. CECP identified 53,690 pairs that contain cross‐boundary alignment in total, but only 22,758 of these met the TM‐score threshold after rearrangement. Notably, the SSE‐duplication pipeline completed the entire dataset in approximately 130 CPU hours, making it over 16,000 times more efficient than CECP. For a detailed comparison see Table 1.

**TABLE 1:** Comparison of SSE‐duplication and CECP on a filtered RCSB dataset.

Metric	SSE duplication	CECP
Overall dataset		
Unique proteins analyzed	17,130	17,130
Cross‐boundary alignment (pairs)	67,984	53,690
Pairs with TM‐score ≥ 0.5	67,984	40,058
Pairs with TM‐score ≥ 0.5 and TM_SSE_–TM_CECP_ ≥ 0.1	67,984	22,758
Pairs identified by both methods		
Highly similar (TM‐score ≥ 0.8)	4433	4663
Pairs where TM_SSE_–TM_CECP_ >0.1	7600	–
Pairs where TM_CECP_–TM_SSE_ >0.1	–	1018
CPs unique to SSE duplication		
Unique SSE‐only CP pairs	14,294	–
Highly similar (TM‐score ≥ 0.8)	236	217
Pairs where TM_SSE_–TM_CECP_ >0.1	4095	–
Pairs where TM_CECP_–TM_SSE_ >0.1	–	660

We further analyzed the results by dividing them into two groups: (a) circular permutations (CPs) identified by both methods, and (b) CPs uniquely identified by the SSE duplication method. For the subset identified by both methods, the SSE duplication pipeline generally recovers substructures with higher overall similarity. The number of highly similar substructures is comparable between the methods (TM‐score≥0.8): 4433 for SSE duplication versus 4663 for CECP. For the same protein pairs, SSE duplication identifies over 7600 pairs with markedly higher similarity than CECP, defined by

$$\Delta {\mathrm{TM}}_{1}={\mathrm{TM}}_{\mathrm{SSE}}-{\mathrm{TM}}_{\text{CECP}}\geq 0.1,$$

whereas CECP exceeds SSE in only 1018 pairs,

$$\Delta {\mathrm{TM}}_{2}={\mathrm{TM}}_{\text{CECP}}-{\mathrm{TM}}_{\mathrm{SSE}}\geq 0.1.$$

For CPs not recognized by CECP, we observed the same trend. SSE duplication continues to identify more highly similar substructures, with 236 cases exceeding a TM‐score of 0.8, compared to 217 for CECP. Again, in terms of relative similarity, SSE identifies 4095 pairs with $\Delta {\mathrm{TM}}_{1}>=0.1$, while CECP identifies only 660 such cases with $\Delta {\mathrm{TM}}_{2}>=0.1$.

Upon visual inspection of several CECP pairs that extracted structurally similar substructures but were not classified as CPs, we found that many involve indel mutations combined with near‐symmetric rearrangements. In particular, we observed cases where a smaller protein aligns well with both a continuous internal region and the disjoint N‐ and C‐termini of a larger protein. More details of these comparisons can be viewed at Table 1 and an example of this phenomenon is shown in Figure S2.

*Comparison with CPDB*—For the benchmark comparison, we applied both the SSE‐duplication method and CECP to reproduce circular‐permutation pairs reported in the Circular Permutation DataBase (CPDB). To ensure a fair comparison for the SSE‐based approach, we excluded any pair in which either protein contains fewer than five secondary‐structure elements (SSEs), yielding a filtered set of 3102 pairs. Within this set, the SSE‐duplication method identified 2410 pairs with cross‐boundary alignments, whereas CECP identified 2853 pairs.

Among the pairs identified by both methods, CECP generally reported higher structural similarity between the extracted substructures; however, the effect size was modest, with an average TM‐score increase of ≈0.047 relative to the SSE‐duplication method, that is,

$$\Delta \mathrm{TM}\;=\;{\mathrm{TM}}_{\text{CECP}}-{\mathrm{TM}}_{\mathrm{SSE}}\;\approx \;0.047.$$

See Figure 3.

**FIGURE 3:**
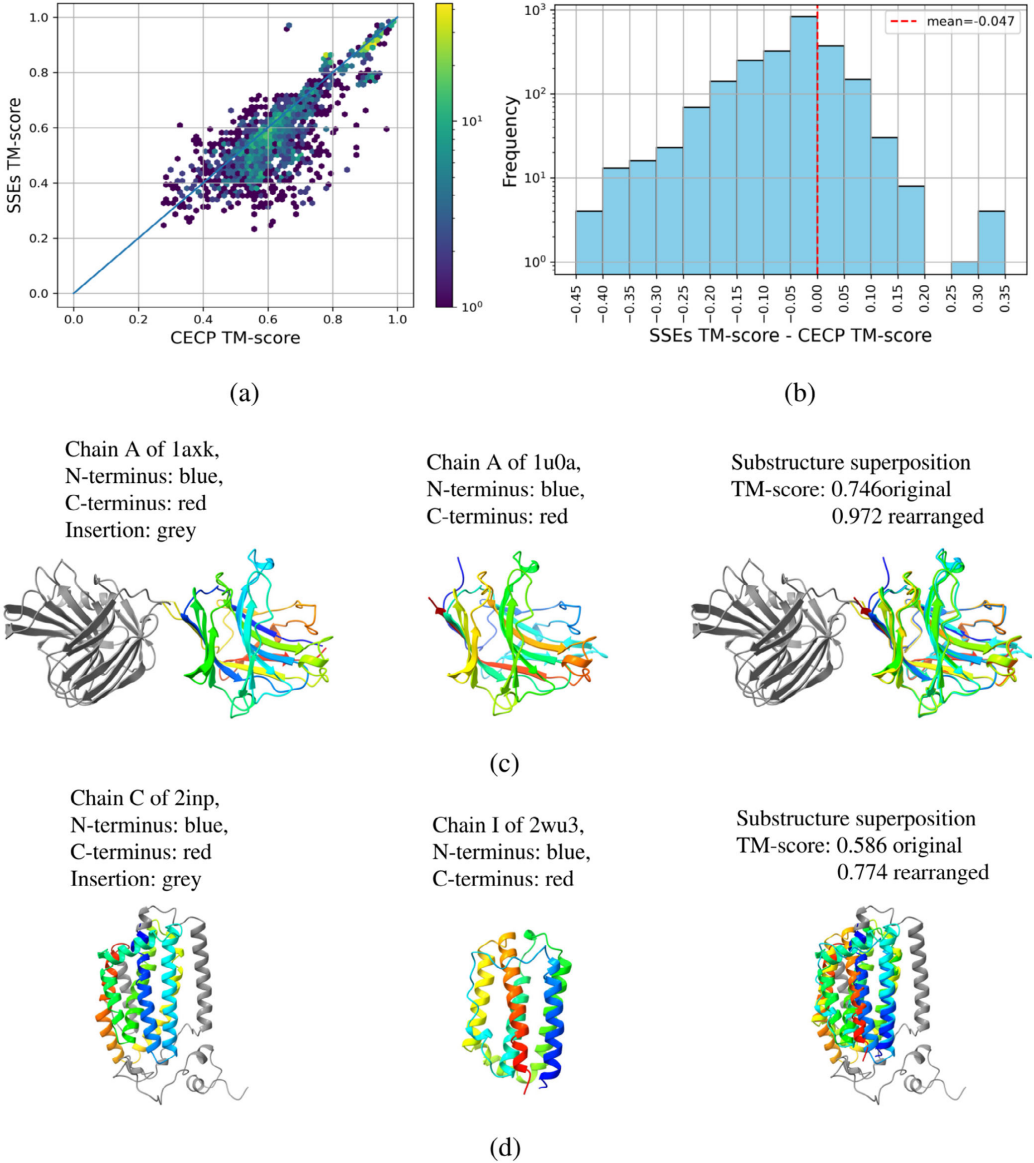
Benchmarking SSE duplication against CECP on the CPDB dataset. (a) Correlation scatter plot of rearranged TM‐scores obtained by SSE duplication versus CECP. (b) Distribution of the differences in rearranged TM‐scores (SSE−CECP). (c) Case study: Advantage of SSE Duplication. Alignment of 1u0a_A and 1axk_A, where SSE duplication achieved a TM‐score of 0.972. While CECP identified the permuted range in 1u0a_A, it failed to account for an insertion in 1axk_A, leading to wrong alignment (TM‐score: 0.661). (d) Case study: Limitation of SSE Duplication. An instance where CECP outperformed SSE duplication. Repetitive helical structures confounded the SSE‐based boundary detection, resulting in misalignment and a low TM‐score of 0.330. Overall, while CECP performs slightly better on average, both methods yield comparable structural similarity scores.

Given the low computational cost of the SSE‐duplication method, we performed exhaustive pairwise comparisons among proteins in the CPDB dataset. This analysis uncovered an additional 1,559 protein pairs that satisfy the criteria for circular permutation but were not previously annotated in CPDB. These newly identified pairs are efficiently detected by the SSE‐duplication method. In contrast, identifying these pairs with CECP would require substantially more computational resources, underscoring the superior scalability and practical sensitivity of our approach.

#### 2.1.2 Indel mutations

As many novel circularly permuted pairs exhibited substantial gaps between their aligned boundaries, we extended our analysis to indel mutations. We found that there are in total 199,804 pairs of proteins that are indel mutants of each other, corresponding to 26,379 unique proteins. Furthermore, 15,966 of the previously identified 67,984 permuted pairs, corresponding to 8855 unique proteins, also harbor indels. These dually modified proteins cluster into 813 groups, highlighting the additional structural diversity introduced by insertions and deletions (Figure 2c and Figure S4). Notably, many fluorescent proteins were identified to contain both circular permutations and indel mutations, which is consistent with the known tolerance of green fluorescent protein (GFP) to both circular permutations and indel mutations (Baird et al., 1999).

#### 2.1.3 Symmetry detection from cross‐boundary alignment

Many hits from the SSE‐duplication pipeline exhibited a TM‐score difference (ΔTM) below 0.1 between the original and permuted orders, and thus were not classified as circular permutants. This small difference suggests that such proteins may possess inherent structural symmetry or exhibit domain‐size imbalance that biases alignments toward the same region. Visual inspection supports this interpretation, revealing frequent symmetric features. To systematically assess symmetry, we split each protein at its duplication boundary and calculated the TM‐score between the resulting segments; proteins with TM≥0.5 were classified as symmetric (Jinrui & Zhang, 2010) (Figures S7 and S9). For comparison, we also applied CE‐Symm (Bliven et al., 2019) to the same non‐redundant dataset, selecting proteins with TM≥0.5 and more than one repeat unit. Although the number of symmetric proteins detected by SSE‐duplication (7226) and CE‐Symm (7108) was similar, the overlap between the two methods was limited, with a Jaccard index of just 0.240. The unique predictions made by each method are illustrated in Figure S1.

Comparing unique predictions highlights complementary strengths:

- *CE‐Symm* excels at detecting proteins whose repeat units have very high structural similarity.
- *SSE‐duplication* uncovers symmetry in proteins with more complex SSE arrangements (see Figure S3).

In general, the unique predictions by these two methods have a very similar distribution of symmetry and number of SSEs. The SSE‐duplication method is currently limited by its reference set for cross‐boundary alignments and support for only two‐fold symmetry.

Together, these results demonstrate that our SSE‐duplication pipeline not only reliably captures standard circular permutations with strong agreement to CPDB and provides a comprehensive view of structural symmetry in proteins, but also uncovers novel, indel‐modified permuted proteins—highlighting its sensitivity and broad applicability to protein structural variation.

### 2.2 Self‐scanning of SSEs

To overcome the limitations of detecting symmetry solely based on cross‐boundary alignment (which targets circular permutations), we employed a scanner‐based self‐alignment strategy using SSEs.

**FIGURE 4:**
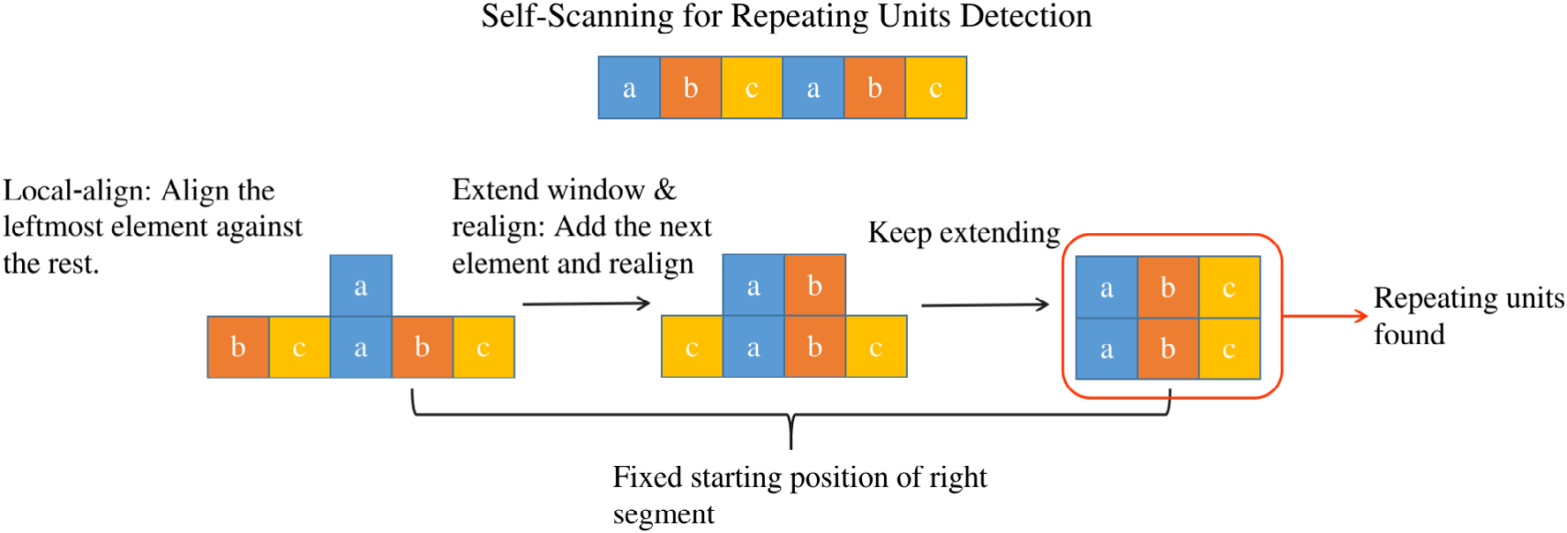
Self‐scanning of SSEs. Starting from the leftmost secondary structure element (SSE) in each sequence, we slide a window that initially contains three SSEs and extend it rightward one SSE at a time until only three SSEs remain at the end. Each extracted segment pair is then verified using TM‐align to assess internal symmetry.

*Efficiency and scalability*: Taking advantage of the compact nature of SSEs, we implemented a sliding window approach (Figure 4). Starting from the N‐terminus, a window of three consecutive SSEs is aligned against the remainder of the chain, extending rightward one SSE at a time. Because RCSB proteins contain on average only ∼19 SSEs, this exhaustive scan entails only ∼14 alignments per protein. Compared with residue‐level scanning methods (e.g., using 3Di or amino acids), this element‐based approach is computationally more efficient and robust against local residue fluctuations.

*Hierarchical unit detection*: This scanning process naturally reveals the hierarchical organization of protein symmetry. We distinguish the hierarchy of repeating units by analyzing the *boundary shifts* between successive optimal alignments. For example, a target start shift of ∼30 SSEs indicates a single‐blade repeat, whereas a shift of ∼60 SSEs indicates a double‐blade repeat and ∼90 SSEs indicates a triple‐blade repeat (as visualized in Figure 6).

*Noise consolidation*: To resolve redundant hits (e.g., slightly shifted or truncated boundaries), we employ a *sequential maximization algorithm*. We process candidates sorted by sequence position and apply a greedy selection strategy: retaining hits with the highest TM‐scores and prioritizing longer sequence spans when scores are comparable. This effectively filters out truncated artifacts in favor of complete, structurally defined units.

**FIGURE 5:**
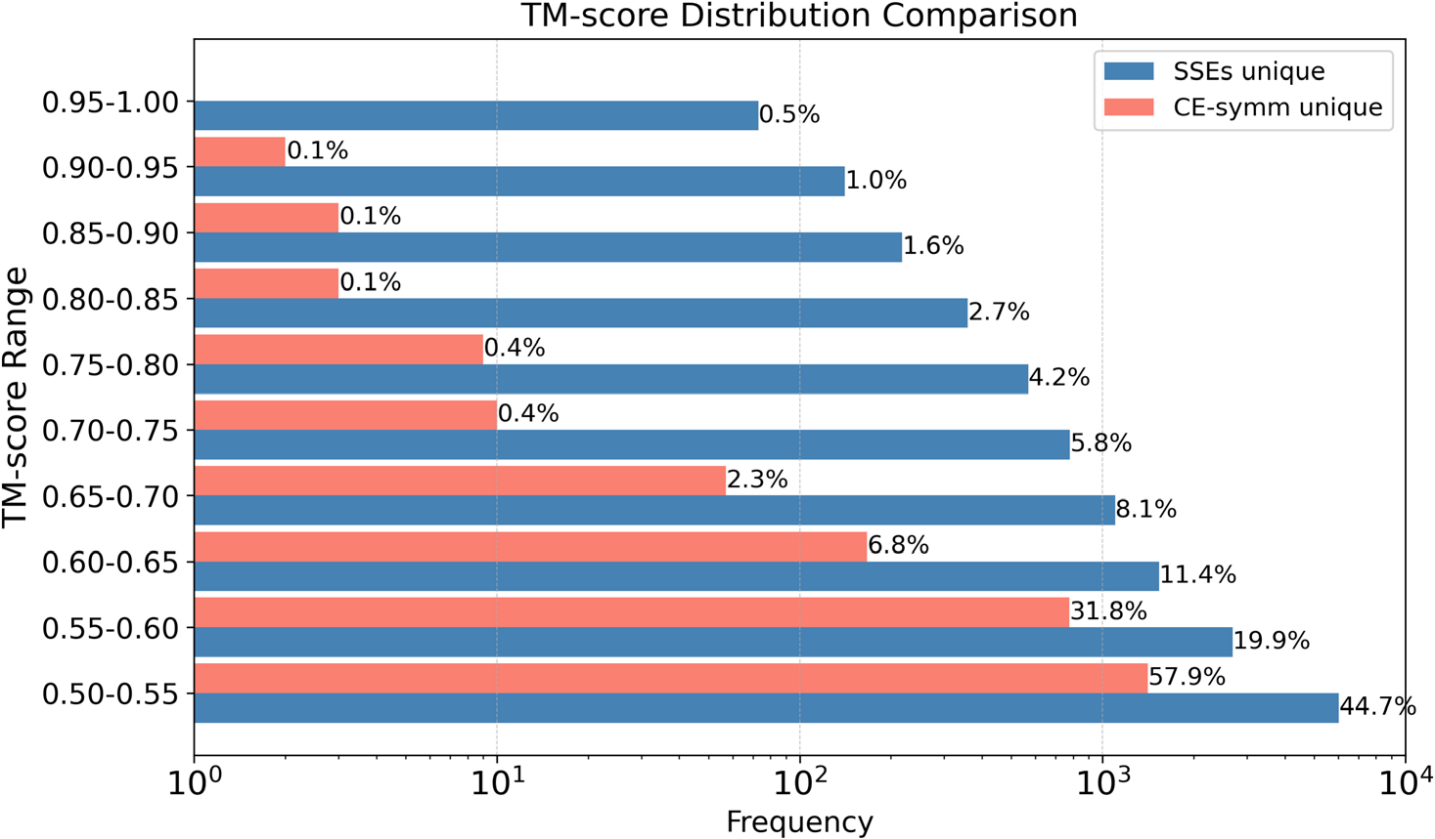
TM‐score distribution of unique predictions. This distribution compares the TM‐scores of unique symmetry protein predictions by the SSE‐based and CE‐Symm methods. Nearly 90% of the CE‐Symm predictions lie between 0.5 and 0.6, suggesting moderate symmetry, while the SSE‐based method identifies a broader range, capturing many highly symmetric structures.

This approach identified 18,173 proteins that contain sub‐structure pairs with TM‐scores above 0.5 (requiring a minimum of 3 SSEs per substructure) and detected more symmetric proteins than the union of those identified by the CE‐Symm and SSE‐based methods for circular permutations. Specifically, this method produced 13,505 unique findings and recovered 65.6% of the symmetric proteins found by CE‐Symm, which had 2440 unique predictions. Comparison of the TM‐scores in the symmetric regions of the unique predictions reveals that nearly 90% of the CE‐Symm unique predictions have TM‐scores below 0.6, whereas only 65% of the SSE‐based unique predictions fall below that threshold. This indicates that CE‐Symm may miss many highly similar, symmetric structures, while the SSE‐based method tends to omit structures with only moderate similarity. (see Figure 5).

**FIGURE 6:**
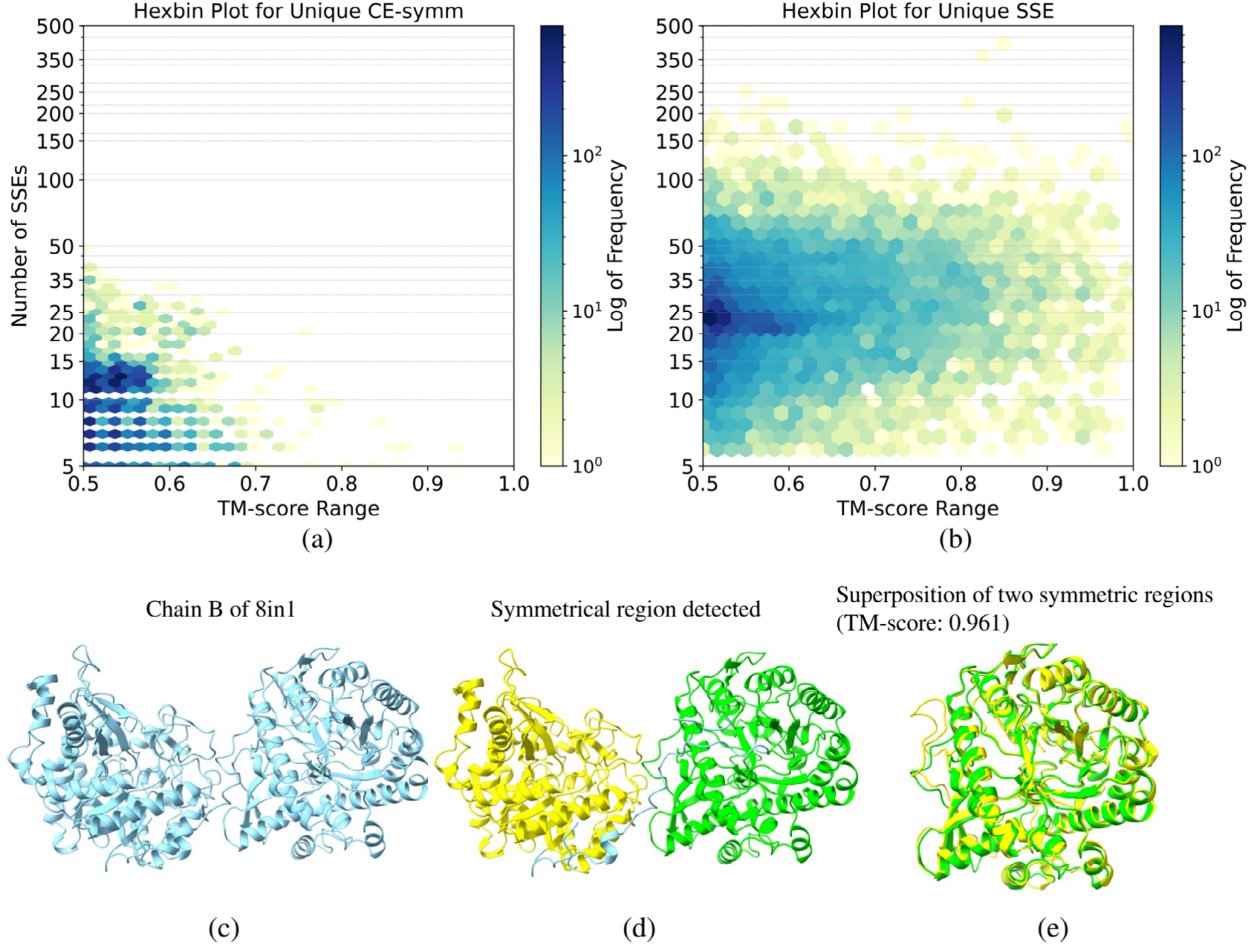
TM‐score and number of SSEs correspondence of unique predictions. (a) TM‐score vs. number of SSEs for proteins uniquely detected by the SSE‐based method. (b) Same for CE‐Symm. (c) A representative example of a large and highly symmetric protein identified by the SSE‐based method but missed by CE‐Symm. These plots show that CE‐Symm fails to detect symmetric regions in large proteins (none with above 50 SSEs), whereas the SSE‐based approach successfully identifies local symmetry even in proteins with up to 500 SSEs, demonstrating superior sensitivity and scalability for detecting structural symmetry.

Furthermore, analysis of the correspondence between the number of SSEs in a protein and the TM‐score of its symmetric region shows that CE‐Symm struggles to capture local symmetry in large proteins—none of its unique predictions contains more than 50 SSEs—whereas the SSE‐based method can detect symmetric regions in proteins with up to 500 SSEs. Many of these large proteins exhibit extremely symmetric structures. These findings highlight the superior sensitivity and scalability of the SSE‐based approach in detecting local structural symmetry. It not only recovers more symmetric proteins overall but also captures highly symmetric regions in large and complex structures that CE‐Symm consistently misses. (See Figure 6).

We further compared the number of repeating units detected by each method across the overlapping set of proteins. In most cases, both methods identified only two symmetric units, and their results were generally consistent. However, significant discrepancies emerged when proteins contained a larger number of repeats. In such cases, CE‐Symm typically identified more repeated units than the SSE‐based method. (See Figure S5).

Visual inspection of these differences revealed that CE‐Symm is more effective at detecting smaller, localized repeating units, particularly in highly symmetric regions. This discrepancy arises from the SSE‐based method's summarization approach, which may overlook smaller units. For example, consider a protein composed of four identical repeats (A‐A‐A‐A). During self‐scanning, the first repeat may align better with the third than with the second. Because the scanning window extends one SSE at a time, the boundaries between units may shift such that the four repeats are grouped into two larger units, rather than being recognized individually. More details on the self‐scanning procedure and how repeated units are summarized are provided in Section 4.7.

## 3 DISCUSSION AND CONCLUSION

In this study, we developed an SSE‐duplication pipeline to effectively detect internal structural features in proteins by aligning across secondary structure element (SSE) boundaries. The pipeline successfully recovered most circular permutations previously annotated in redundant RCSB entries and the CPDB, additionally identifying 1559 previously unannotated pairs within CPDB. In total, we detected 67,984 circularly permuted pairs across 17,130 unique proteins out of 71,781 analyzed. A recognized limitation is that proteins with few SSEs or a TM‐score below 0.6 might evade detection due to weakened alignment signals from sparse SSE representations.

Significantly, our analysis revealed a notable intersection between circular permutations and insertion–deletion (indel) mutations. Among the analyzed proteins, 26,379 proteins exhibited indel mutations, comprising 199,804 unique indel‐mutant pairs. Strikingly, 8855 proteins exhibited both circular permutations and indel mutations, emphasizing a potential co‐evolutionary relationship between these two structural variations. Network analysis further revealed highly skewed cluster‐size distributions in both the full circular permutation dataset and the subset containing indel mutations, reinforcing the hypothesis of co‐evolutionary mechanisms linking circular permutation and indel events.

To assess internal symmetry, we compared the results of cross‐boundary SSE alignments against the established CE‐Symm method. Although both methods yielded similar counts (7226 proteins for SSE duplication versus 7108 for CE‐Symm), the overlap was limited (Jaccard index = 0.24), indicating complementary strengths. CE‐Symm excels at identifying small, highly similar repeat units, whereas our SSE‐duplication method covers a broader range of SSE counts but remains inherently restricted to detecting twofold symmetry and relies on boundary crossings or database matches.

Despite the success of our SSE‐duplication approach, it has inherent constraints. The method requires sufficiently long SSEs and cannot resolve repeats involving more than two units. Additionally, the reduction of full protein structures to SSEs may obscure flexible loops and small helices, thus reducing sensitivity in regions with sparse or short SSEs. Future enhancements could involve incorporating additional structural features, such as backbone geometry or loop conformations, into the SSE representation. An adaptive substitution matrix implemented via a neural network could further improve circular permutation boundary detection, though both strategies would increase computational complexity.

To address limitations inherent to SSE duplication for internal symmetry detection, we developed a self‐scanning SSE approach specifically tailored for this purpose. This method significantly outperformed CE‐Symm, identifying 18,173 symmetric proteins and covering 65.6% of CE‐Symm's predictions. Missed cases were primarily associated with proteins containing few SSEs or moderate structural similarity.

Further analysis indicated that the SSE‐based method surpasses CE‐Symm not only in the number of detected symmetric proteins but also in handling structurally complex proteins. Our approach successfully identified symmetry in proteins containing up to 500 SSEs, whereas CE‐Symm's capability is restricted to fewer than 50 SSEs.

Regarding repeated unit identification, both methods generally agreed, though CE‐Symm demonstrated greater proficiency in capturing spatially independent units. This discrepancy arises from differing methodologies in defining and summarizing repeats, as detailed in the Methods section.

Additionally, CE‐Symm uniquely distinguishes between open symmetry (linear or helical repeats) and closed symmetry (cyclic or looped arrangements), a feature not supported by our SSE‐based approach. Nevertheless, the SSE‐duplication pipeline offers significant advantages in computational efficiency, scalability, and sensitivity, providing robust opportunities for advanced protein structural analysis and protein engineering applications.

## 4 METHOD

### 4.1 Extraction and representation of secondary structure elements (SSEs)

To derive compact Secondary Structure Elements (SSEs), secondary structure assignments were first computed from atomic coordinates using the DSSP algorithm (Scheme 2) (Joosten et al., 2011). The standard 8‐state DSSP output was reduced to three simplified classes: α‐helix (“H”), β‐strand (“E”), and random coil (“C”).

To isolate the topological core, random coils were discarded. The remaining sequence was compressed into discrete tokens representing contiguous segments of “H” or “E.” We employed a variable‐resolution discretization scheme based on segment length to handle the wide variance in SSE sizes:

1. *Exact mapping*: Short segments (lengths 2–10) are mapped to unique character tokens (e.g., length 2 → “A,” length 3 → “B”).
2. *Compressed mapping*: To mitigate sparsity in the vocabulary, longer segments are binned. Lengths 12–30 are mapped with a step size of 2, while segments with lengths >30 are mapped with a step size of 3.

Crucially, the secondary structure type is encoded via case sensitivity to distinguish topological identity: helical segments are assigned lowercase tokens (e.g., HHH → “b”), whereas β‐strands are assigned uppercase tokens (e.g., EEE → “B”). The complete implementation of this preprocessing pipeline is available in the associated GitHub repository (Lin & Ahnert, 2025).

### 4.2 Database construction and preprocessing

The experimental dataset was constructed by integrating protein structural data from two primary repositories: the RCSB Protein Data Bank (PDB) (Burley et al., 2024). The data retrieval cutoff dates were set to May 3rd, 2024, for the PDB. To ensure statistical independence and mitigate homology bias during evaluation, the PDB dataset underwent redundancy reduction using a 90% sequence identity threshold.

### 4.3 Implementation of SSE alignment and scoring

Secondary structure assignments were derived using the DSSP algorithm (Joosten et al., 2011) (as defined in Section 4.1). For topological comparison, pairwise alignment of SSE sequences was performed utilizing the Smith–Waterman algorithm with element‐specific gap penalties. The substitution matrix employed in this alignment was rigorously optimized via a genetic algorithm, specifically targeted to maximize the recovery rate of SCOPe domains (Fox et al., 2013). Subsequently, high‐dimensional features derived from the alignment—including sequence identity and length coverage—were processed by a hybrid ResNet–BiLSTM architecture to regress TM‐scores. A detailed exposition of the machine learning framework and the optimization pipeline for SSE‐based structure searching is provided in our previous work (Lin & Ahnert, 2025).

### 4.4 Duplication of SSEs

Circular permutation and indel mutations are determined by cross‐boundary alignments, achieved by duplicating the query SSEs and aligning them to the original target SSEs. Illustration of these alignments can be found at Figure 2a. Alignments crossing the original protein boundary are filtered by a predicted TM‐score threshold of 0.5 and further verified by actual TM‐score calculations. Circular permutations with indel mutations are defined as cases where a gap exists between cross‐boundary segments. This gap is typically defined as at least 40 residues (approximately three SSEs, based on the requirement of at least three SSEs per side) or at least 60% of the length of the smaller segment in terms of residues. Indels of 20–30 residues are generally treated as minor loop variations (Triant & Pearson, 2015). Symmetry is determined if cross‐boundary aligned segments achieve a TM‐score above 0.5 (Jinrui & Zhang, 2010).

### 4.5 TM‐align

TM‐align (Zhang & Skolnick, 2005) is a pairwise protein‐structure alignment algorithm that uses only the C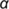 coordinates of two protein chains to compute a normalized TM‐score (Zhang & Skolnick, 2004) between 0 and 1. A TM‐score above 0.5 generally indicates that two structures share the same fold (Jinrui & Zhang, 2010).

For circular permutation analysis, two proteins with identical C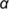 coordinates and the same number of residues in each permuted segment will yield a TM‐score of approximately 0.5 in the original sequence order, rising to 1.0 after correctly rearranging the permuted units. In practice, the pre‐ and post‐rearrangement TM‐scores depend not only on the overall structural similarity but also on the relative size of the largest permuted segment.

### 4.6 Symmetry proteins by CE‐Symm

CE‐Symm is a dedicated tool for detecting internal symmetry in proteins (Bliven et al., 2019). It applies the Combinatorial Extension (CE) algorithm to align a protein with a copy of itself while forbidding near‐diagonal alignments to avoid trivial matches. In this study, we consider symmetric segments with TM‐scores above 0.5 with more than 1 repeated unit as symmetric proteins.

### 4.7 Self‐scanning of SSEs for symmetry detection

**FIGURE 7:**
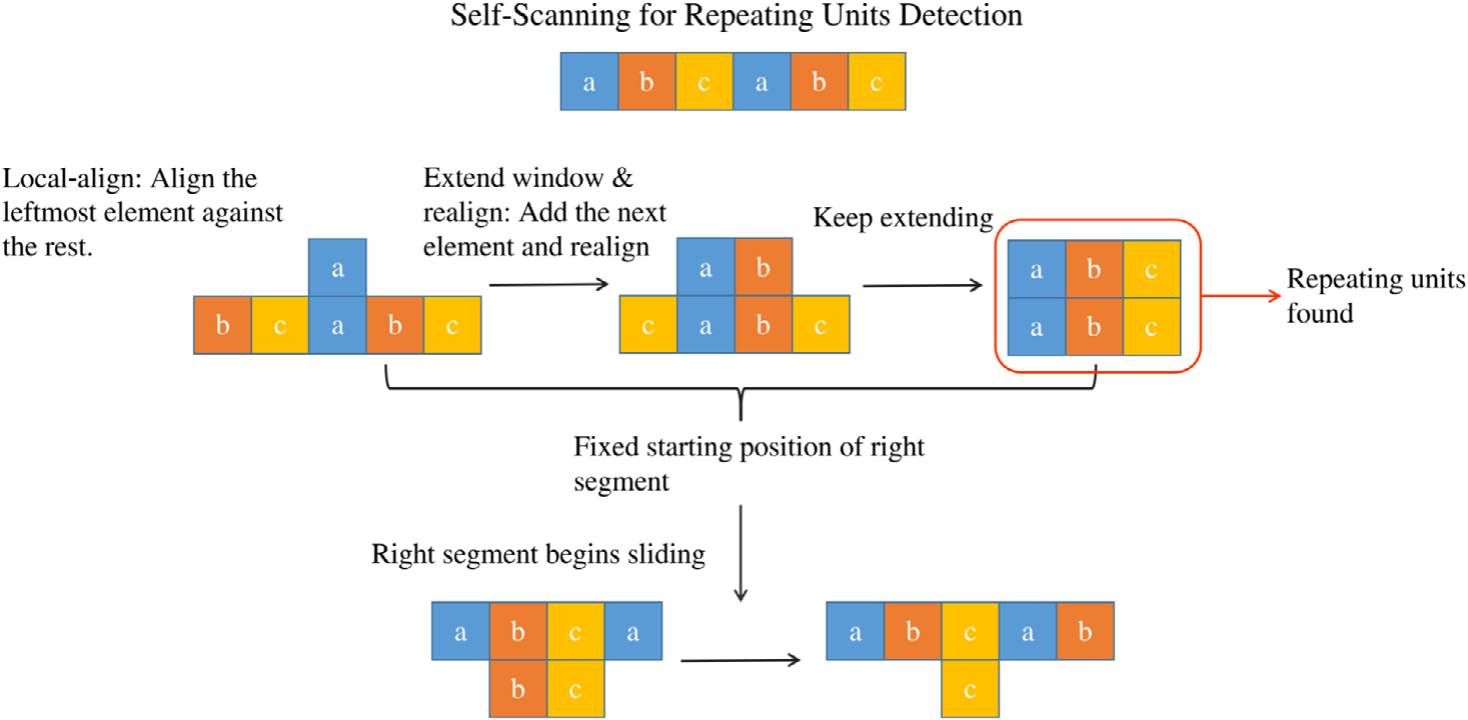
Self‐scanning for repeating units detection. Illustration of the self‐scanning method for detecting repeating structural units. The input sequence is scanned by fixing the starting position of the left segment, extending it one element at a time, and aligning it against the right segment. When an optimal alignment is found (highlighted), repeating units are identified. After all fixed positions of the right segment are exhausted, the right segment begins to shift, continuing the scan.

To detect symmetric proteins, the self‐scanning strategy described in the Results was implemented with specific filtering criteria. Further implementation details are provided in Algorithm 1 (Figure 7).

ALGORITHM 1 **Self‐scanning SSE repeat detection.**

1: **Input**: list of $\left(\mathrm{pdb},{\mathrm{rng}}_{1},{\mathrm{rng}}_{2},\text{score}\right)$ tuples

2: **Output**: total repeat count per PDB

3: // 1. Group by PDB

4: lines_by_pdb← map PDB → list of tuples

5: // 2. Build & merge clusters

6: **for all** pdb in lines_by_pdb **do**

7: G← buildGraph($\text{lines}\_\mathrm{by}\_\mathrm{pdb}\left[\mathrm{pdb}\right]$).

8: clusters← mergeComponents(connectedComponents(G))

9: store clusters in $\text{clusters}\_\mathrm{by}\_\mathrm{pdb}\left[\mathrm{pdb}\right]$

10: **end for**

11: // 3. Split & summarize repeats

12: **for all** $\left(\mathrm{pdb},\;\text{clusters}\right)$ in clusters_by_pdb **do**

13: total_repeats←0

14: **for all** cluster in clusters **do**

15:  subs← splitOnGaps(cluster,3).

16:  **for all** sub in subs **do**

17:   $\left(b,s,r\right)\leftarrow$ summarize(sub)

18:   total_repeats+=r

19:  **end for**

20: **end for**

21: record total_repeats for pdb

22: **end for**

23: **return** repeat counts per PDB

*Sliding window parameters*: The scan initiates with a window size of 3 SSEs (the minimum required for a valid topological unit) and extends iteratively. The iteration stops when fewer than three SSEs remain in the unaligned portion of the chain.

*Filtering and consolidation details*: Since the procedure generates multiple candidate alignments, we first apply a global filter for TM‐score >0.5. The *sequential maximization algorithm* is implemented as follows:

1. Candidates are sorted by their sequence start position.
2. For overlapping candidates, we calculate the TM‐score difference (ΔTM).
3. If ΔTM≥0.05, the candidate with the higher score is retained.
4. If ΔTM<0.05, the candidate with the longer sequence span (total residue coverage) is retained.

Finally, to determine the symmetry order, we construct a graph where nodes represent the consolidated aligned segments and edges connect segments with high structural similarity. We extract connected components and compute the repeat count for each component as its total boundary coverage divided by its minimal unit length.

## AUTHOR CONTRIBUTIONS

**Runfeng Lin:** Investigation; validation; visualization; writing – original draft; formal analysis; software; methodology; data curation. **Sebastian Ahnert**: conceptualization; methodology; supervision; writing.

## Supporting information


**Figure S1.** Symmetric Proteins Identified by SSEs and CE‐Symm. (a) A protein uniquely identified as symmetric by the SSE‐based method, displaying a TM‐score of 0.914. (b) A protein uniquely identified by CE‐Symm, with a TM‐score of 0.960. These examples demonstrate that while both methods capture a comparable number of symmetric proteins, each uniquely detects distinct local symmetry features.
**Figure S2.** Example of a circular permutation detected by SSE duplication but not by CECP, with similar TM‐scores. (a) Chain A of PDB 6r5z, colored from blue at the C‐terminus to red at the N‐terminus; (b) Chain E of PDB 7w5a, colored from blue at the C‐terminus to red at the N‐terminus; (c) Superposition using the SSE‐duplication–derived boundary; (d) Superposition using the CECP‐derived boundary. Both them show very well matched which means symmetry is a special type of Circular Permutations.
**Figure S3.** Comparison of Unique Predictions by SSEs and CE‐Symm. (a) TM‐score distribution of unique predictions from SSEs and CE‐Symm. Both methods show a similar distribution, with CE‐Symm identifying slightly more symmetric proteins when the symmetric units are highly similar. (b) Distribution of the number of SSEs in unique predictions. CE‐Symm captures more symmetry in smaller proteins, while SSEs are more effective in identifying symmetry in larger proteins. These results suggest that CE‐Symm and SSEs capture complementary aspects of structural symmetry.
**Figure S4.** Structural Clustering of Circular Permutants and Indel Mutants. (a) Example of circular permutation. (b) Example of circular permutation with indel mutation. These examples demonstrate the effectiveness of the SSE‐based approach in detecting structural similarity in circular permutations and the impact of indel mutations on clustering.
**Figure S5.** Comparison of repeated units identified by SSE‐based and CE‐Symm methods. (a) Comparison of the number of repeats identified by SSEs and CE‐Symm. (b) Comparison of average TM‐scores of symmetric units from both methods. (c, d) Symmetric units in chain A of 6tjg: CE‐Symm identifies 8‐fold while SSEs identifies 2‐fold. (e, f) Symmetric units in chain A of 3p6j: CE‐Symm identifies 3‐fold while SSEs identifies 4‐fold. Overall, the two methods generally agree, though CE‐Symm tends to find more symmetric units.
**Figure S6.** Correlation between TM‐score and sequence identity improvement in circularly permuted proteins. The mean increase in TM‐score was 0.143, compared to a marginal increase of 0.022 for sequence identity. Although a minimum TM‐score increment of 0.1 was applied as the threshold for defining circular permutations, the corresponding sequence identity did not exhibit a proportional rise. This discrepancy is expected, as protein structure is significantly more conserved than sequence, highlighting the superior sensitivity of structure‐based detection methods.
**Figure S7.** Correlation between TM‐score and sequence identity for symmetric units in proteins. Symmetric units were defined using a minimum TM‐score threshold of 0.5, with a requirement of at least three secondary structure elements (SSEs) per unit. The identified symmetric units exhibit a mean TM‐score of 0.616, whereas their mean sequence identity is only 0.208. Contextually, analyses of the CATH database indicate that protein domains sharing a TM‐score of 0.5 belong to the same fold in approximately 37% of cases, rising to 80% when the TM‐score reaches 0.6 (Jumper et al., 2021; Wang et al., 2024). These results highlight the advantage of our structure‐based method over sequence‐based approaches in identifying evolutionarily related but sequentially divergent structures.
**Figure S8.** Comparison of TM‐score and sequence identity in circularly permuted proteins versus a shuffled SSE null model. (a) Correlation between the TM‐scores of the original and rearranged structures. (b) Correlation between the rearranged TM‐score and the TM‐score of structures with shuffled Secondary Structure Elements (SSEs). The comparison of (a) and (b) demonstrates that while the local 3D coordinates of the segments remain identical, the specific connectivity of the SSEs is crucial for global structural similarity. (c) Correlation between TM‐score and sequence identity for the shuffled structures. Notably, although the low sequence identity suggests no evolutionary relationship, a subset of shuffled structures still exhibits high structural similarity, highlighting the independence of fold from sequence in these generated topologies.
**Figure S9.** Comparison of TM‐score and sequence identity in symmetric proteins versus a shuffled SSE null model. (a) Correlation between the TM‐scores of the shuffled SSEs and the original symmetric units. (b) Correlation between TM‐score and sequence identity for the native symmetric units. (c) Correlation between TM‐score and sequence identity for the shuffled structures. While shuffling generally disrupts the global fold—evidenced by the large fraction of proteins degrading into structural noise—the shuffled dataset surprisingly contains slightly more high‐similarity structures than the circularly permuted (CP) dataset. This observation aligns with theories of algorithmic probability and simplicity bias in the genotype‐phenotype map: symmetric topologies appear to be intrinsically likely and “easy to find” in the structural landscape, potentially emerging spontaneously due to their low descriptive complexity rather than solely through selective pressure (Bliven et al., 2014; Lo et al., 2008).

## Data Availability

The source code for the SSE‐based structural analysis pipeline is publicly available on GitHub at https://github.com/rl647/SSE_search and data are available at https://github.com/rl647/SSE_search/tree/main/data.
