# Supplementary material for: Detection of protein symmetry and structural rearrangements using secondary structure elements
Source: Protein Sci. 2026 Apr 30;35(6):e70576. doi: 10.1002/pro.70576 (PMC13133429; doi:10.1002/pro.70576)
Supplement: Supplementary file 1 — Figure S1. Symmetric Proteins Identified by SSEs and CE‐Symm. (a) A protein uniquely identified as symmetric by the SSE‐based method, displaying a TM‐score of 0.914. (b) A protein uniquely identified by CE‐Symm, with a TM‐score of 0.960. These examples demonstrate that while both methods capture a comparable number of symmetric proteins, each uniquely detects distinct local symmetry features. Figure S2. Example of a circular permutation detected by SSE duplication but not by CECP, with similar TM‐scores. (a) Chain A of PDB 6r5z, colored from blue at the C‐terminus to red at the N‐terminus; (b) Chain E of PDB 7w5a, colored from blue at the C‐terminus to red at the N‐terminus; (c) Superposition using the SSE‐duplication–derived boundary; (d) Superposition using the CECP‐derived boundary. Both them show very well matched which means symmetry is a special type of Circular Permutations. Figure S3. Comparison of Unique Predictions by SSEs and CE‐Symm. (a) TM‐score distribution of unique predictions from SSEs and CE‐Symm. Both methods show a similar distribution, with CE‐Symm identifying slightly more symmetric proteins when the symmetric units are highly similar. (b) Distribution of the number of SSEs in unique predictions. CE‐Symm captures more symmetry in smaller proteins, while SSEs are more effective in identifying symmetry in larger proteins. These results suggest that CE‐Symm and SSEs capture complementary aspects of structural symmetry. Figure S4. Structural Clustering of Circular Permutants and Indel Mutants. (a) Example of circular permutation. (b) Example of circular permutation with indel mutation. These examples demonstrate the effectiveness of the SSE‐based approach in detecting structural similarity in circular permutations and the impact of indel mutations on clustering. Figure S5. Comparison of repeated units identified by SSE‐based and CE‐Symm methods. (a) Comparison of the number of repeats identified by SSEs and CE‐Symm. (b) Comparison of average TM‐sco [file PRO-35-e70576-s001.pdf]

Supplementary Materials

Detection of Protein Symmetry and Structural Rearrangements using Secondary Structure Elements

Runfeng Lin[1] and Sebastian E. Ahnert[1,2,3]

[1]Theory of Condensed Matter Group, Cavendish Laboratory, University of Cambridge, JJ Thomson Avenue, Cambridge CB3 0HE, UK
[2]Department of Chemical Engineering and Biotechnology, University of Cambridge, Philippa Fawcett Drive, Cambridge CB3 0AS, UK
[3]The Alan Turing Institute, 96 Euston Road, London NW1 2DB, UK

Contents

- Supplementary Figures

S1 Supplementary Figures

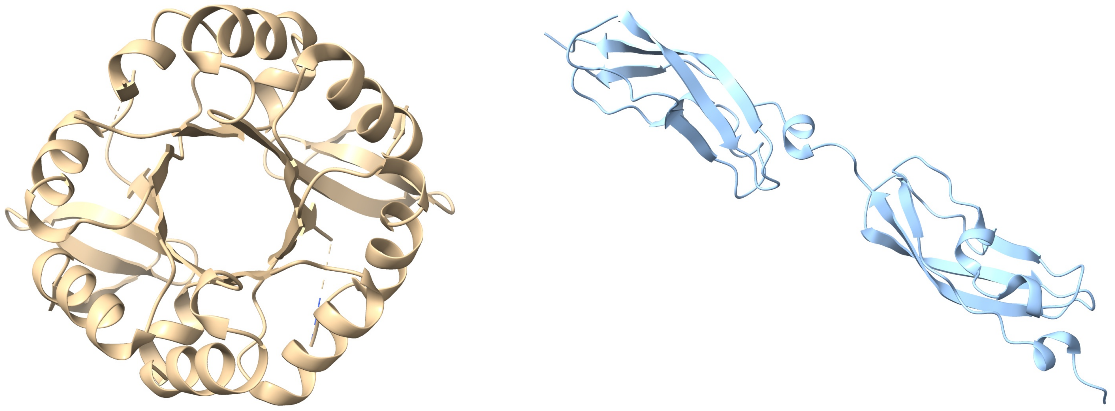

(a) (b)

Figure S1: **Symmetric Proteins Identified by SSEs and CE-Symm.** (a) A protein uniquely identified as symmetric by the SSE-based method, displaying a TM-score of 0.914. (b) A protein uniquely identified by CE-Symm, with a TM-score of 0.960. These examples demonstrate that while both methods capture a comparable number of symmetric proteins, each uniquely detects distinct local symmetry features.

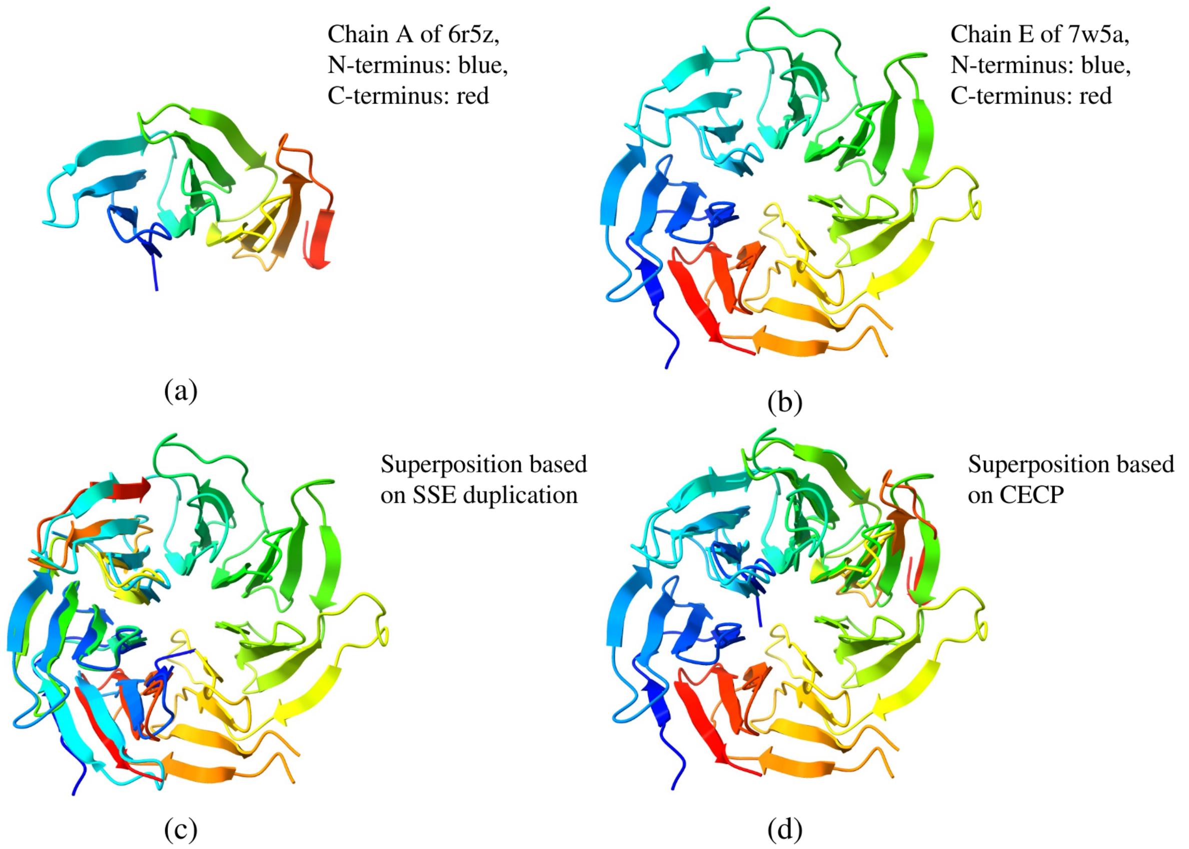

Figure S2: **Example of a circular permutation detected by SSE duplication but not by CECP, with similar TM-scores.** (a) Chain A of PDB 6r5z, colored from blue at the C-terminus to red at the N-terminus; (b) Chain E of PDB 7w5a, colored from blue at the C-terminus to red at the N-terminus; (c) Superposition using the SSE-duplication–derived boundary; (d) Superposition using the CECP-derived boundary. Both them show very well matched which means symmetry is a special type of Circular Permutations.

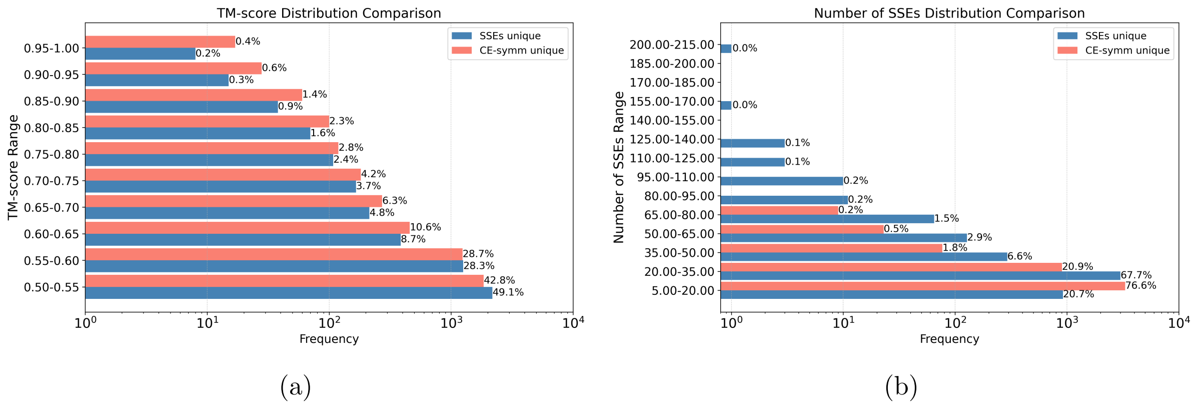

Figure S3: **Comparison of Unique Predictions by SSEs and CE-Symm.** (a) TM-score distribution of unique predictions from SSEs and CE-Symm. Both methods show a similar distribution, with CE-Symm identifying slightly more symmetric proteins when the symmetric units are highly similar. (b) Distribution of the number of SSEs in unique predictions. CE-Symm captures more symmetry in smaller proteins, while SSEs are more effective in identifying symmetry in larger proteins. These results suggest that CE-Symm and SSEs capture complementary aspects of structural symmetry.

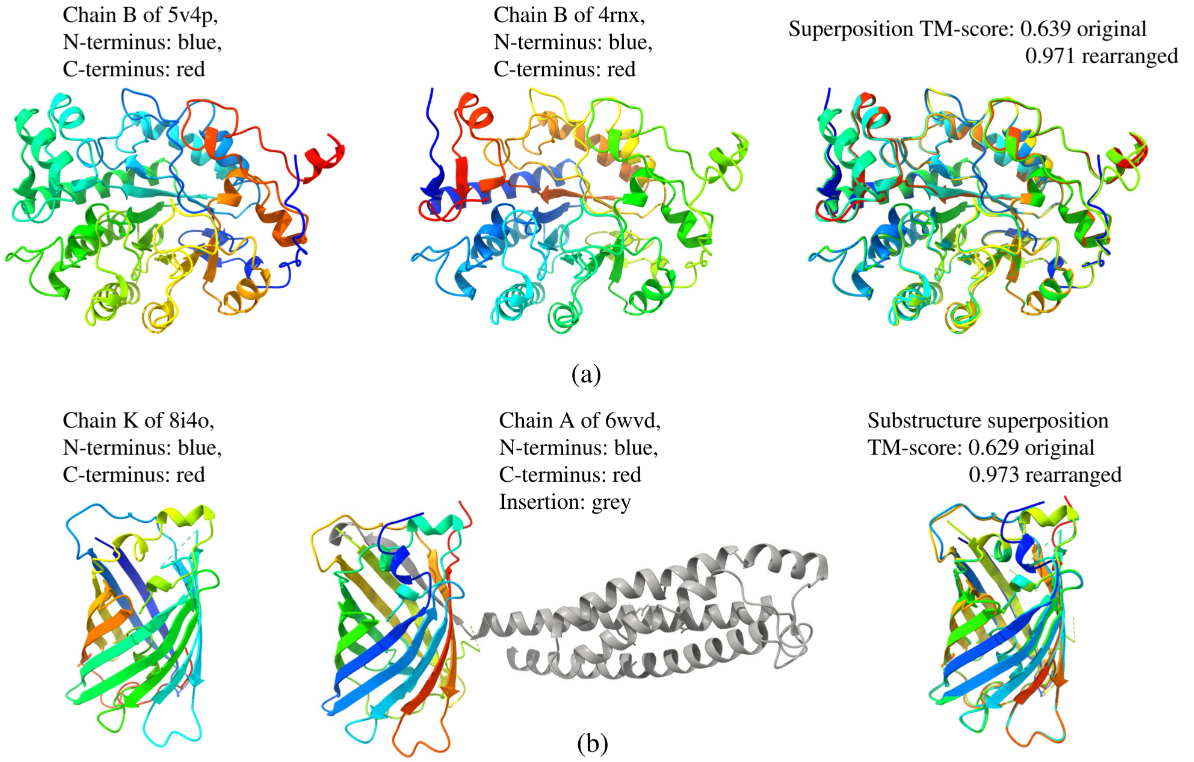

Figure S4: **Structural Clustering of Circular Permutants and Indel Mutants.** (a) Example of circular permutation. (b) Example of circular permutation with indel mutation. These examples demonstrate the effectiveness of the SSE-based approach in detecting structural similarity in circular permutations and the impact of indel mutations on clustering.

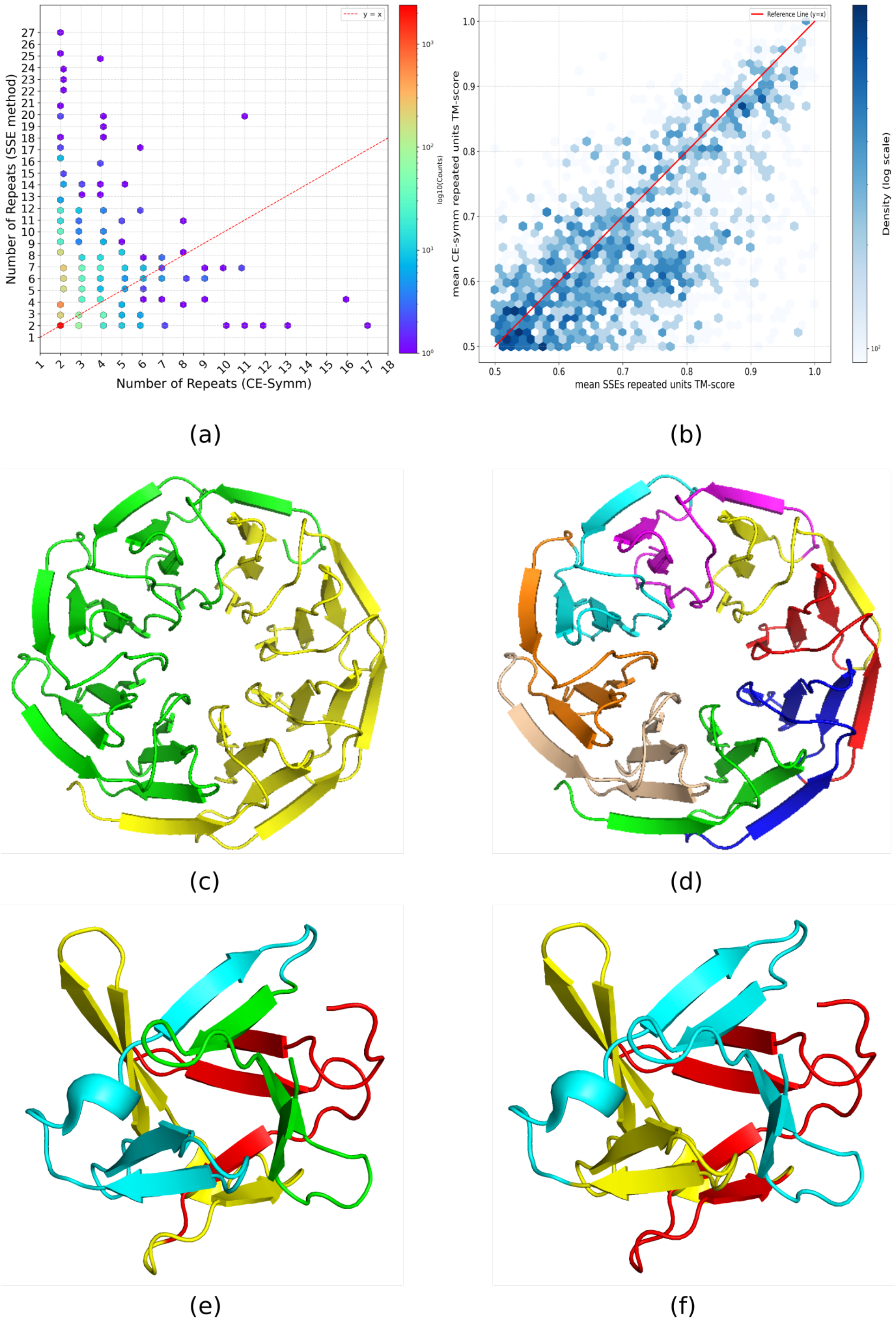

Figure S5: **Comparison of repeated units identified by SSE-based and CE-Symm methods.** (a) Comparison of the number of repeats identified by SSEs and CE-Symm. (b) Comparison of average TM-scores of symmetric units from both methods. (c, d) Symmetric units in chain A of 6tjg: CE-Symm identifies 8-fold whilst SSEs identifies 2-fold. (e, f) Symmetric units in chain A of 3p6j: CE-Symm identifies 3-fold whilst SSEs identifies 4-fold. Overall, the two methods generally agree, though CE-Symm tends to find more symmetric units.

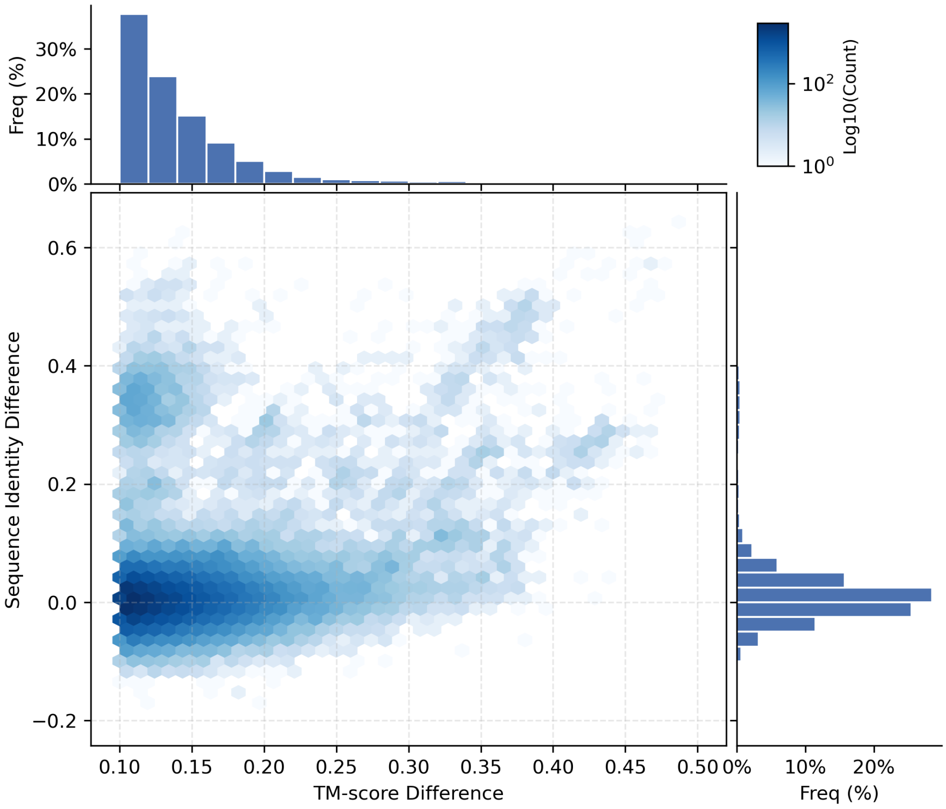

Figure S6: **Correlation between TM-score and sequence identity improvement in circularly permuted proteins.** The mean increase in TM-score was 0.143, compared to a marginal increase of 0.022 for sequence identity. Although a minimum TM-score increment of 0.1 was applied as the threshold for defining circular permutations, the corresponding sequence identity did not exhibit a proportional rise. This discrepancy is expected, as protein structure is significantly more conserved than sequence, highlighting the superior sensitivity of structure-based detection methods.

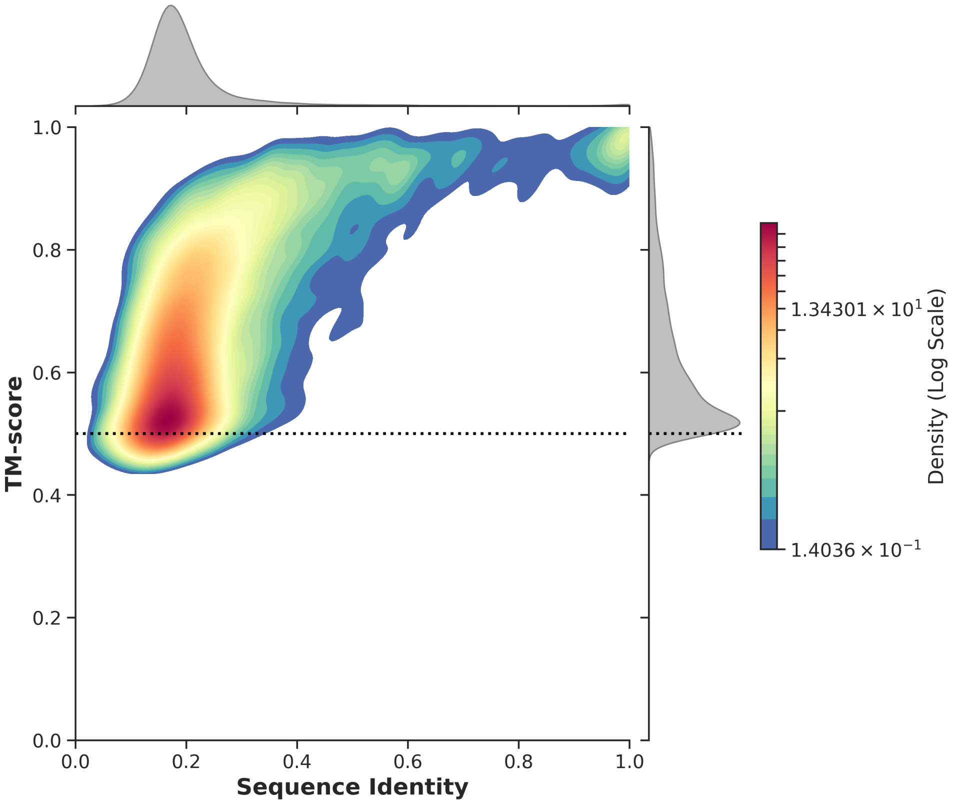

Figure S7: **Correlation between TM-score and sequence identity for symmetric units in proteins.** Symmetric units were defined using a minimum TM-score threshold of 0.5, with a requirement of at least three secondary structure elements (SSEs) per unit. The identified symmetric units exhibit a mean TM-score of 0.616, whereas their mean sequence identity is only 0.208. Contextually, analyses of the CATH database indicate that protein domains sharing a TM-score of 0.5 belong to the same fold in approximately 37% of cases, rising to 80% when the TM-score reaches 0.6 [1, 2]. These results highlight the advantage of our structure-based method over sequence-based approaches in identifying evolutionarily related but sequentially divergent structures.

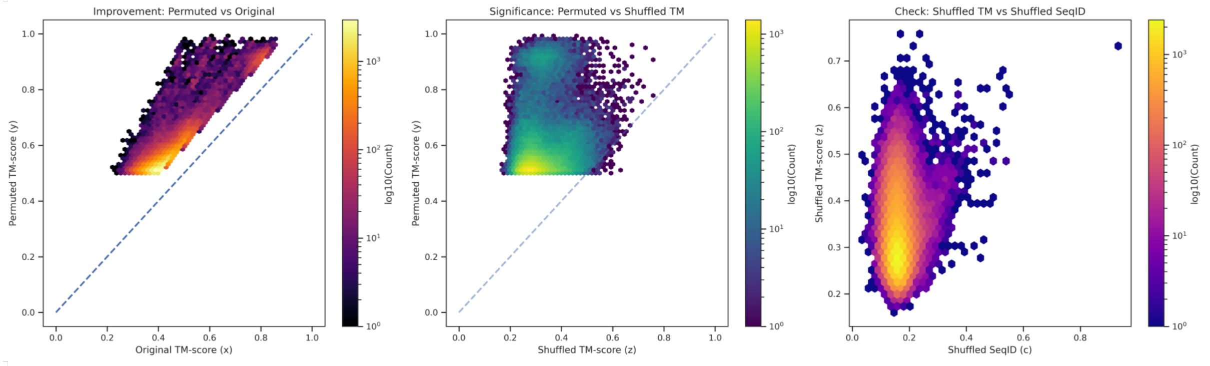

(a) (b) (c)

Figure S8: **Comparison of TM-score and sequence identity in circularly permuted proteins versus a shuffled SSE null model.** (a) Correlation between the TM-scores of the original and rearranged structures. (b) Correlation between the rearranged TM-score and the TM-score of structures with shuffled Secondary Structure Elements (SSEs). The comparison of (a) and (b) demonstrates that while the local 3D coordinates of the segments remain identical, the specific connectivity of the SSEs is crucial for global structural similarity. (c) Correlation between TM-score and sequence identity for the shuffled structures. Notably, although the low sequence identity suggests no evolutionary relationship, a subset of shuffled structures still exhibits high structural similarity, highlighting the independence of fold from sequence in these generated topologies.

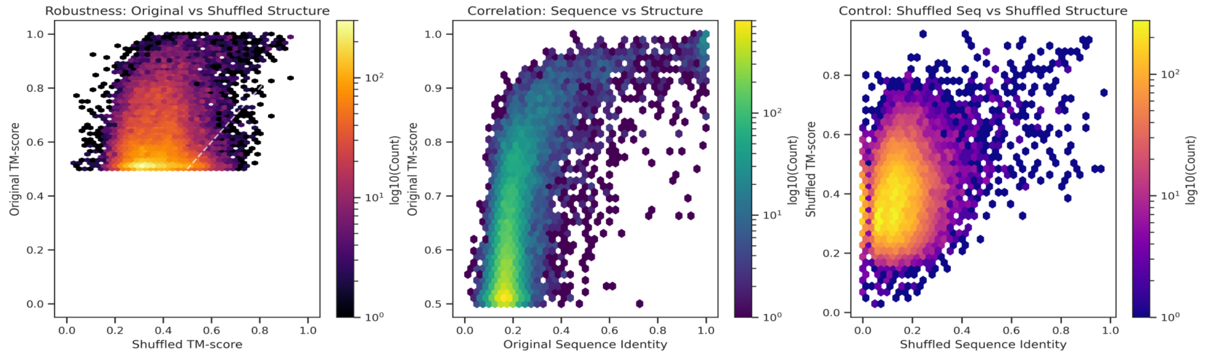

(a) (b) (c)

Figure S9: **Comparison of TM-score and sequence identity in symmetric proteins versus a shuffled SSE null model.** (a) Correlation between the TM-scores of the shuffled SSEs and the original symmetric units. (b) Correlation between TM-score and sequence identity for the native symmetric units. (c) Correlation between TM-score and sequence identity for the shuffled structures. While shuffling generally disrupts the global fold—evidenced by the large fraction of proteins degrading into structural noise—the shuffled dataset surprisingly contains slightly more high-similarity structures than the circularly permuted (CP) dataset. This observation aligns with theories of **algorithmic probability** and **simplicity bias** in the genotype-phenotype map: symmetric topologies appear to be intrinsically likely and "easy to find" in the structural landscape, potentially emerging spontaneously due to their low descriptive complexity rather than solely through selective pressure [3, 4].

References

[1] Jinrui Xu and Yang Zhang. How significant is a protein structure similarity with tm-score = 0.5? *Bioinformatics*, 26(7):889–895, February 2010.

[2] Vaishali P Waman, Nicola Bordin, Andy Lau, Shaun Kandathil, Jude Wells, David Miller, Sameer Velankar, David T Jones, Ian Sillitoe, and Christine Orengo. Cath v4.4: major expansion of cath by experimental and predicted structural data. *Nucleic Acids Research*, 53(D1):D348–D355, November 2024.

[3] Sebastian E. Ahnert. Structural properties of genotype–phenotype maps. *J. R. Soc. Interface*, 14, 2017.

[4] Iain G. Johnston, Kamaludin Dingle, Sam F. Greenbury, Chico Q. Camargo, Jonathan P. K. Doye, Sebastian E. Ahnert, and Ard A. Louis. Symmetry and simplicity spontaneously emerge from the algorithmic nature of evolution. *Proceedings of the National Academy of Sciences*, 119(11), March 2022.
